# Current Perspectives on the Role of Collagen Peptide Fragments in Health and Disease

**DOI:** 10.3390/cells15181626

**Published:** 2026-09-08

**Authors:** Kenzi-Alayna Campbell, Sanjay P. Tirupattur, Gia Katwa, Laxmansa C. Katwa

**Affiliations:** Department of Physiology, Brody School of Medicine at East Carolina University, Greenville, NC 27834, USAtirupatturs24@students.ecu.edu (S.P.T.); katwag26@students.ecu.edu (G.K.)

**Keywords:** collagen peptide fragments, extracellular matrix, degradation, cardiovascular disease, heart failure

## Abstract

Collagen is the most abundant protein in the human body and a vital component of the extracellular matrix (ECM), where it maintains structural architecture, strength, and mechanical stability throughout the body. Collagen undergoes continuous turnover and remodeling mediated by matrix metalloproteinases (MMPs) and other proteases to generate collagen peptide fragments (CPFs). CPFs were long considered inert byproducts of collagen turnover but are now recognized to have two overlapping roles: as circulating and urinary biomarkers of ECM remodeling, and as beneficial or adverse bioactive matrikines that directly regulate inflammation, angiogenesis, fibrosis, immune responses, and tissue remodeling. This review focuses on current evidence regarding the biological activities of various CPFs across cardiovascular, cancer, and pulmonary systems. We conclude by discussing various perspectives, including clinical and therapeutic applications and future directions.

## 1. Introduction

Collagen is the most abundant structural protein in the human body and is distributed as a major structural component of connective tissues, basement membranes, and specialized extracellular matrices in tissues such as skin, bone, cartilage, tendon, blood vessels, heart, lungs, liver, and kidneys [1,2]. Collagens are primarily produced by fibroblasts and deposited into the ECM. There are 28 known collagen types in vertebrates, with collagen type I (Col I) being the most abundant, accounting for 90% of collagen. Collagens are classified according to the organization of their triple-helical collagenous domains and supramolecular assembly into fibril-forming collagens, fibril-associated collagens with interrupted triple helices (FACIT), network-forming collagens, beaded-filament-forming collagens, and anchoring fibrils [3]. Each collagen type varies in gene expression and alpha chain composition, resulting in structurally and functionally distinct collagen molecules. Although fibrillar procollagens contain N- and C-terminal propeptides that are removed during maturation, this architecture should not be generalized to all collagen families.

**Figure 1:**
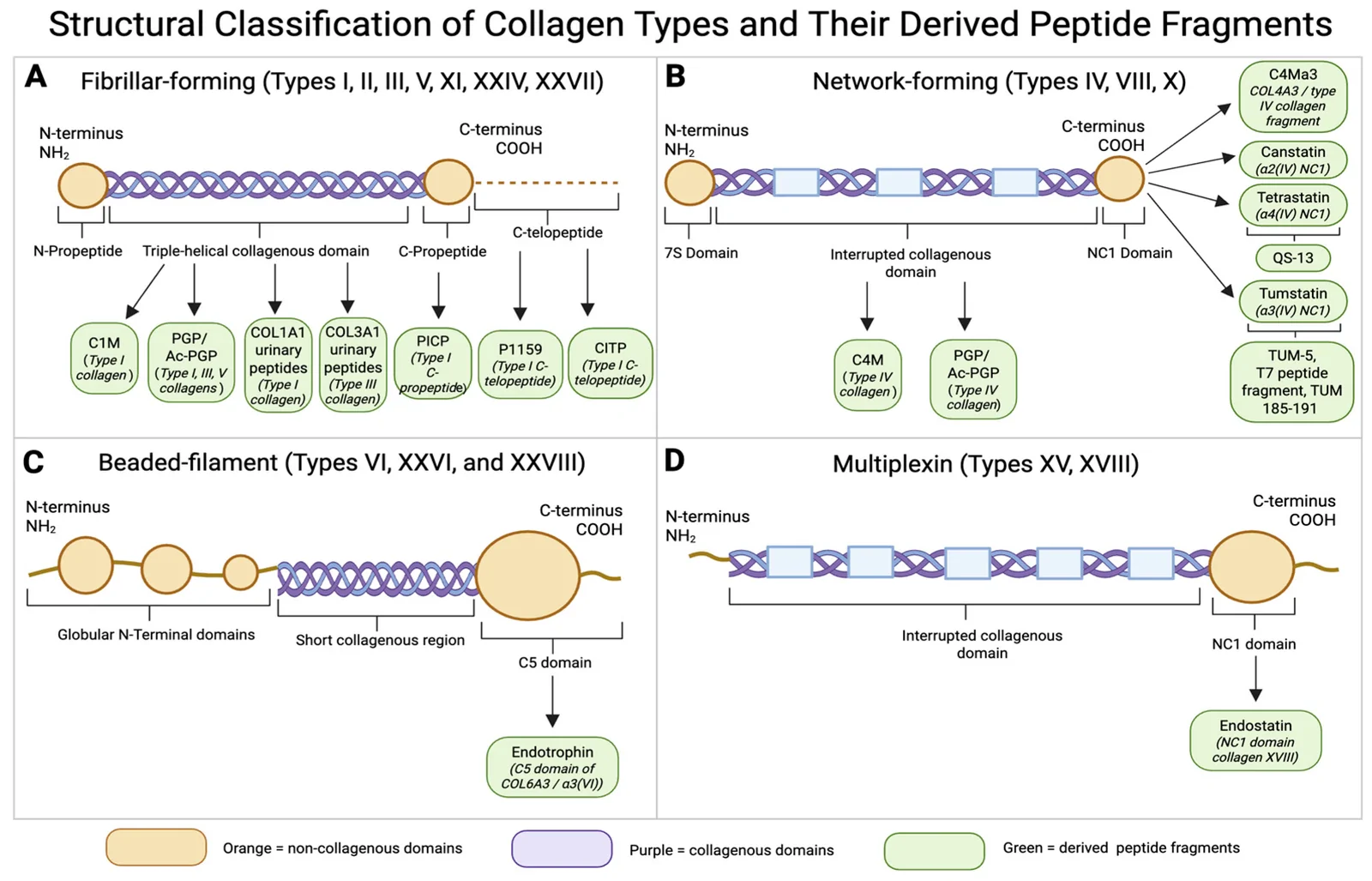
**Representative collagen-derived peptide fragments discussed in this review and their structural origins**. Selected collagen types are shown to illustrate the domain architectures from which peptide fragments discussed in this manuscript are generated [3]. (**A**) In fibrillar procollagen (Types I and III), proteolytic processing of propeptides, triple-helical region, and telopeptides generates fragments including PICP, C1M, P1159, and CITP [1,4]. (**B**) In network-forming collagen (Type IV), cleavage of the NC1 domain generates the matrikines arresten, canstatin, tumstatin, and tetrastatin from the α1(IV), α2(IV), α3(IV), and α4(IV) chains, respectively; QS-13 is a bioactive peptide derived from tetrastatin [1]. (**C**,**D**) In collagen VI, cleavage of the C5 domain of the α3(VI)/COL6A3 chain releases endotrophin, whereas cleavage of the NC1 domain of collagen XVIII releases endostatin [1,5]. The collagen types and fragments portrayed in the figure are based on those mentioned in this review and do not entail all known collagen-derived peptides.

Collagen plays crucial physiological roles such as maintaining the structural integrity, strength, and elasticity of connective tissues [2,3]. The structural diversity of collagen families influences their proteolytic processing and the origins of the resulting peptide fragments [1,3,4,5]. Figure 1 summarizes representative architectures of collagens I, III, IV, VI, and XVIII and identifies the structural domains from which selected bioactive and biomarker CPFs discussed in this review are derived. Collagen function is attributed to its defining structural feature, a triple helix, which is formed by three polypeptide α chains containing Gly-X-Y amino acid chain repeats [6,7,8]. Non-collagenous (NC1) domains lack this repeated chain sequence and instead form globular flexible regions [7]. In fibrillar procollagens, the central triple helix is flanked by N and C-terminal propeptides that are enzymatically cleaved during collagen fibril maturation.

MMPs, particularly collagenases (MMP-1, MMP-8, MMP-13) and gelatinases (MMP-2 and MMP-9), are a class of zinc-containing proteases that are directly involved in the degradation of mature collagen into fragments [1,9]. Fibrillar collagen (Col I, Col III, Col V) cleavage is a multi-step process; collagenases first recognize and bind to active exosites on the triple helix, unwind it, and mediate site-specific cleavage of the helix’s native fibrillar collagen [3,9]. Interstitial collagenases such as MMP-1, MMP-8, and MMP-13 cleave the triple helix at a locus approximately three-quarters of the distance from the N-terminus. This cleavage produces an N-terminal ~¾ fragment and a C-terminal ~¼ fragment, which can degrade and undergo further proteolysis by gelatinases to produce peptides with physiological functions [2,9]. Non-fibrillar collagens such as collagen VI (Col VI) and collagen XVIII (Col XVIII) can be cleaved to generate bioactive fragments.

Recently, collagen fragments have been recognized for their potential roles in health and disease. Collagen fragments can serve as biomarkers, holding prognostic or diagnostic value. Some MMP-cleaved collagen fragments are bioactive and commonly referred to as matrikines; these fragments can directly influence biological functions. These classifications are not mutually exclusive as some CPFs can both serve as biomarkers and exhibit bioactive properties in health and disease.

**Figure 2:**
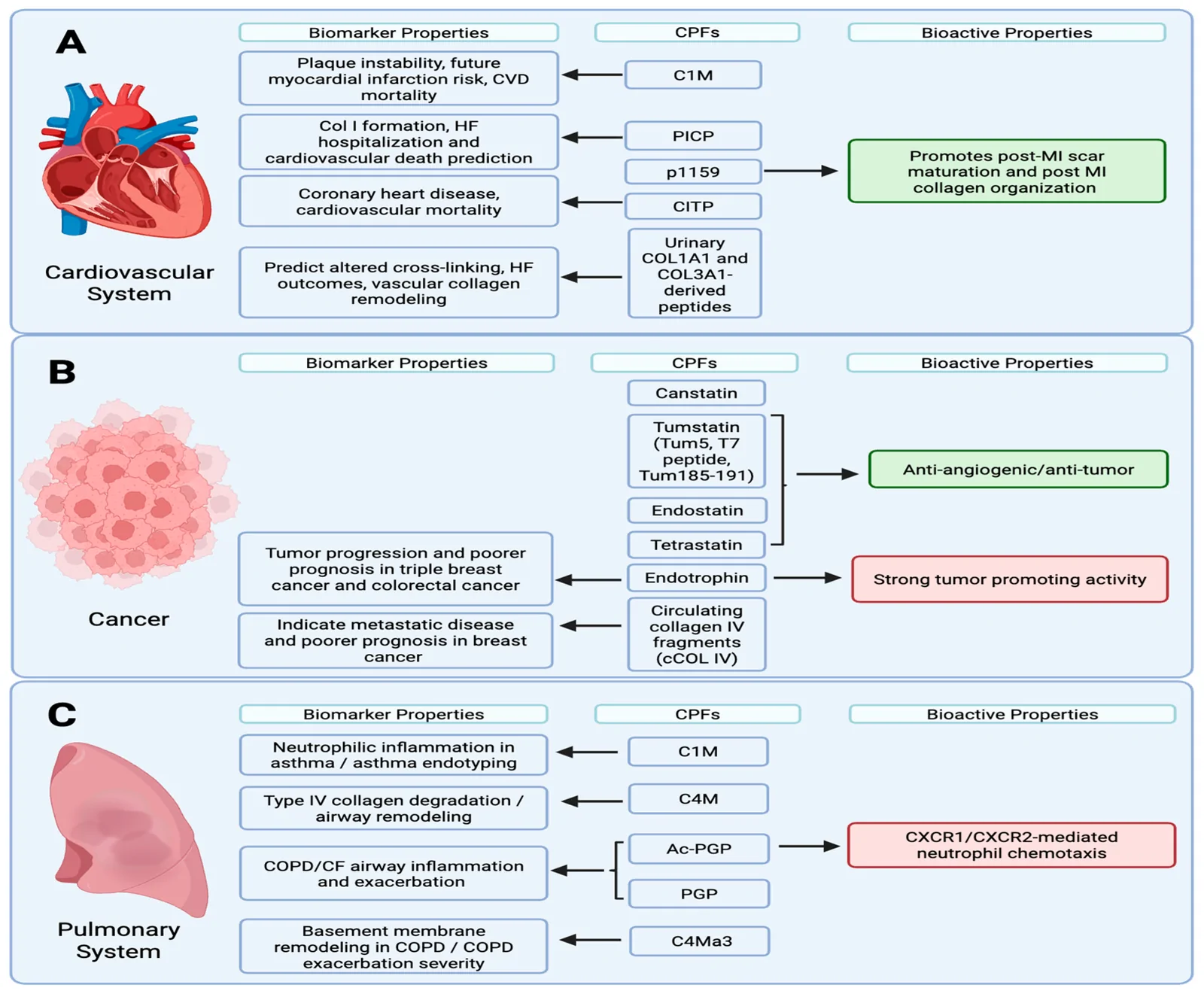
**Influence of peptide fragments derived from collagens across cardiovascular, cancer, and pulmonary systems.** Biomarker properties (**left**) and bioactive properties (**right**) are shown for each CPF. (**A**) In the cardiovascular system, C1M, PICP, CITP, and urinary COL1A1/COL3A1 peptides reflect plaque instability, Col I turnover, and vascular remodeling [10,11,12,13]. P1159 promotes post-MI scar maturation [14]. (**B**) In cancer, canstatin, tumstatin, endostatin, and tetrastatin exhibit anti-angiogenic/anti-tumor activity [1,15]. Endotrophin may promote tumor activity, and circulating collagen IV fragments indicate metastatic disease [1,5,16]. (**C**) In the pulmonary system, C1M is associated with neutrophilic inflammation [17]. C4M and C4Ma3 reflect airway and basement remodeling, while Ac-PGP/PGP drive CXCR1/CXCR2-mediated neutrophil chemotaxis in COPD inflammation [18,19,20].

For the purposes of this review, the term CPFs is broadly used to encompass peptide fragments derived from procollagen processing and proteolytic degradation of mature collagen. CPFs can therefore be distinguished by both their origin and their biological function. The following sections will explore the roles of CPFs in cardiovascular, cancer, and pulmonary systems, as depicted in Figure 2.

## 2. The Effects of CPFs in the Cardiovascular System

Over the past several decades, cardiovascular disease (CVD) has remained the leading cause of death worldwide [21]. An imbalance between collagen synthesis and degradation is regarded as a primary driver of CVD, HF, and cardiac stiffness; maintaining this balance is therefore essential for proper cardiac function [4,22]. In response to CVD and other cardiac pathologies, cardiomyocytes undergo profound pathological remodeling and hypertrophy, leading to impaired function [23]. Within the myocardial ECM, the bulk of cardiac collagen comprises Col I and Col III. Col I constitutes the majority of collagen in cardiac fibroblast-derived ECM and confers the tensile strength required to maintain heart structure, whereas Col III forms an elastic network to confer elastic resilience [2]. As discussed in our previous review, maintaining the ratio between Col I and Col III is essential for normal ECM structure and cardiac function [4]. Given the significance of this balance in maintaining cardiac integrity, the fragments produced by Col I synthesis and degradation have emerged as proxies for monitoring and remodeling processes that underlie cardiac dysfunction.

Among type I collagen-derived CPFs, procollagen type I carboxy-terminal propeptide (PICP) and the carboxy-terminal telopeptide of type I collagen (CITP) provide complementary information regarding collagen turnover. During Col I maturation, the C-terminal propeptide extension is cleaved off from procollagen I, releasing PICP into circulation. PICP is released during collagen maturation, and circulating PICP is commonly used as an indirect blood biomarker of Col I synthesis [11]. In CVD, PICP has been shown to be a strong biomarker of myocardial fibrosis burden, left atrial enlargement, and increased ventricular stiffness, whereas lowering PICP levels is associated with improved cardiac function [24]. PICP also carries prognostic weight, serving as the strongest collagen marker for predicting HF hospitalization and cardiovascular death [24].

In contrast, CITP is generated from MMP-1-mediated hydrolysis of Col I fibrils, making it a biomarker of Col I degradation within the ECM [12]. Specifically, CITP is an established biomarker for tracking collagen deposition linked to plaque vulnerability and cardiac remodeling associated with acute myocardial infarction (MI). Elevated CITP levels can predict worse outcomes in coronary artery disease (CAD) and are associated with the greatest risk for HF hospitalizations and rehospitalizations, preexisting fibrosis, hypertension, and left ventricular remodeling [25]. In elderly patients with HF with reduced ejection fraction (HFrEF), elevated CITP was independently associated with long-term all-cause and cardiovascular mortality in multivariable Cox regression analyses [26].

PICP and CITP reflect distinct aspects of type I collagen turnover. PICP is released in a 1:1 stoichiometric relationship with type I collagen formation during collagen maturation, whereas CITP is released in proportion to the MMP-mediated degradation of mature type I collagen, also exhibiting a 1:1 stoichiometric relationship with collagen breakdown [2,11]. Hence, the PICP:CITP ratio can be used as an index of the relative balance between type I collagen formation and degradation, with higher or lower values reflecting collagen accumulation or degradation, respectively [26]. The PICP and CITP fragments circulate systemically and therefore may reflect collagen turnover in multiple tissues; consequently, the PICP:CITP ratio cannot be interpreted as a cardiac-specific measure of fibrosis. In addition, the CITP:MMP-1 ratio can estimate the quality of myocardial collagen cross-linking, serving as an indirect marker of fiber quality rather than overall turnover. A low CITP:MMP-1 ratio is indicative of extensive cross-linking in hypertensive heart failure, suggesting collagen remains relatively undegraded despite the presence of MMP-1, and is associated with more frequent HF hospitalizations [24]. Low values of both ratios are associated with poor outcomes, and yet CITP sits on opposite sides of the two fractions, and each ratio captures distinct processes in different contexts: a low PICP:CITP ratio tracks mortality in HFrEF by measuring net loss of the collagen scaffold, whereas a low CITP:MMP-1 ratio tracks hospitalization in hypertensive HF by reflecting a stiffened, highly cross-linked matrix [2].

Matrix metalloproteinase-degraded type I collagen (C1M) serves as a biomarker of future myocardial infarction (MI) risk. In response to the inflammation driven by atherosclerosis, macrophages express MMPs, which cleave Col I within the fibrous cap of the plaque and release C1M into the bloodstream. Weaker fibrous caps are more prone to rupture, triggering clot formation that can occlude the coronary artery, increasing the risk of MI. Elevated C1M is therefore indicative of plaque remodeling, making it a biomarker of plaque instability and a risk predictor of MI [10]. Supporting C1M’s prognostic value, Holm Nielsen et al. conducted a study measuring serum C1M in 787 carotid endarterectomy patients, 473 of whom were followed for six years. Results showed that elevated C1M predicted cardiovascular events, cardiovascular mortality, and all-cause mortality [10]. Similarly, in postmenopausal women, C1M in the highest quartile was independently associated with a 33% increased risk of future acute MI relative to the lowest quartile, further supporting C1M as a biomarker of pathological ECM remodeling and cardiovascular disease activity [27]. Since these indices are derived from circulating fragments in blood reflecting systemic collagen turnover, the search for a less invasive alternative has drawn attention to collagen fragments found in the urine.

Urinary collagen fragments are the most abundant urinary peptides and arise from proteolysis of collagen [13]. These fragments are first released into the bloodstream, filtered in the kidneys, and then excreted in the urine [28]. Because urinary collagen fragment patterns mirror collagen turnover in the heart, they serve as a noninvasive biomarker for fibrotic cardiac pathologies. While fibrosis is attributed to excessive collagen deposition in the ECM, urinary fragment patterns implicate attenuated degradation as a major, yet underappreciated driver—cross-linked collagen is resistant to MMPs, reflecting impaired breakdown rather than increased synthesis [13]. This mechanism is demonstrated by the fragments themselves in clinical studies. In a cohort of 354 patients with suspected HF, those who died showed significantly fewer fragments from the rigid central section of collagen type I alpha 1 chain (COL1A1) than from its flexible terminal ends, indicating that the central section resists degradation due to cross-linking. Beyond localizing the specific region of the molecule involved in fibrosis, this signal carried prognostic value: a urinary classifier, built from all HF-associated fragments, predicted an eightfold higher incidence of HF death in the top quartile than in the bottom quartile (20.3% vs. 2.53%), independent of N-terminal pro-B-type natriuretic peptide (Nt-proBNP) [29]. Additionally, in a cohort of 2876 individuals without end-organ damage, classified as hypertensive or normotensive, 68 collagen-derived urinary peptides from vessel wall fibrillar collagens were identified. Among these, COL1A1 and collagen type III alpha 1 chain (COL3A1) fragments were reduced in hypertensive individuals, indicating attenuated collagen degradation due to cross-linking. Because this signal appears prior to any organ damage, these fragments have potential as early markers, possibly reflecting vascular collagen remodeling that precedes cardiovascular disease [30].

Typically, physiological changes following MI include suppressed cell signaling between cardiomyocytes due to excessive fibrotic buildup, leading to cardiac dysfunction. However, as mentioned in Figure 2, CPFs may play an integral role in stabilizing fibrotic scarring following an MI. A study conducted by Grilo et al. investigated the role of p1159 in post-MI fibrotic scar formation [14,31,32]. Notably, p1159 improved scar maturation and promoted the parallel alignment of collagen fibers relative to the epicardium, rather than attenuating interstitial fibrosis post-MI. Additionally, p1159 promoted Col III deposition and reduced expression of lysyl oxidase (LOX2)-mediated cross-linking in females, in turn enhancing vessel perfusion in the infarct zone. Utilizing p1159 in this context provides a different therapeutic axis for post-MI care by steering the repair process, focusing on scar architecture rather than overall limiting fibrosis. Collectively, these collagen fragments demonstrate clinical value in measuring fibrosis, plaque instability, myocardial injury, and patient prognosis rather than being mere byproducts of cardiovascular remodeling.

## 3. The Effects of CPFs on Cancer Progression

Cancer is the second leading cause of death in the United States, and the global cancer burden is predicted to increase by 47% between 2020 and 2040 [33,34]. Cancer-associated fibroblasts (CAFs) are central cell types of the tumor microenvironment (TME), depositing collagen as the principal source of ECM that constitutes a substantial fraction of tumor mass [34,35]. As the most abundant ECM protein in tumors, collagen governs matrix stiffness and forms a physical barrier in the TME that impedes immune cell infiltration; its accumulation initiates a self-reinforcing positive feedback loop that drives further deposition [36]. This process can influence tumor progression, local invasion, and metastatic potential [34,35]. Collagen fragments generated by TME turnover can either promote or restrain tumor progression [34,35].

Within the TME, solid tumors outgrow their vascular supply and give rise to hypoxic regions that stabilize hypoxia-inducible factors (HIF-1α and HIF-2α), driving upregulation of pro-angiogenic factors such as vascular endothelial growth factor (VEGF) [37]. Angiogenesis is consequently regarded as a hallmark of cancer, delivering the oxygen and nutrients required for tumor growth and development [38]. Countering this process, several proteolytically generated fragments derived from the NC1 domains of Col IV and Col XVIII exhibit anti-angiogenic properties. Endostatin, canstatin, and tumstatin all suppress angiogenic processes; however, their receptor interactions and downstream signaling mechanisms are distinct [39]. Endostatin primarily inhibits endothelial cell migration through integrin-associated signaling, whereas tumstatin inhibits endothelial cell proliferation and protein synthesis through integrin alpha-V beta-3 (αvβ3)-dependent suppression of the focal adhesion kinase/phosphoinositide 3-kinase/protein kinase B/mechanistic target of rapamycin (FAK/PI3K/Akt/mTOR) pathway. Canstatin interacts with αvβ3 and αvβ5 integrins and promotes apoptosis in endothelial and tumor cells. These distinct mechanisms and additional anti-tumor activities are discussed below [40].

Endostatin is a natural 20 kDa fragment of the C-terminal NC1 domain of collagen XVIII and is characterized as an endogenous anti-angiogenic inhibitor. Endostatin exhibits anti-angiogenic activity coupled with active immune remodeling in the TME [39,41,42]. This dual vascular immune mechanism recurs across various types of cancer. In triple-negative breast cancer, an endostatin-EGFR fusion protein inhibited vasculogenic mimicry and capillary-like tube formation in endothelial cells, reducing primary tumor growth and metastasis [40]. In a Lewis lung carcinoma model, endostatin not only reduced angiogenesis but may also have made the TME less immunosuppressive by reducing pro-tumor M2 macrophages while increasing cytotoxic cluster of differentiation 8 (CD8+) T cells, allowing destruction of malignant cells [43]. Likewise, in a Human Colon Tumor 116 (HCT-116) colorectal model, endostatin reduced markers associated with angiogenesis such as VEGF, Platelet Endothelial Cell Adhesion Molecule-1 (CD31), and HIF-1α. Endostatin also normalized colorectal tumor vessels, potentially improving CD8+ T cell entry [40].

Canstatin is an anti-angiogenic matrikine derived from the NC1 domain of the α2 chain of Col IV and inhibits endothelial cell survival through suppression of FAK-PI3K-Akt signaling and induction of apoptosis [37]. Canstatin amplifies Fas ligand expression and activates apoptotic pathways in endothelial cells, further contributing to its anti-angiogenic activity [44]. Canstatin also exhibits direct anti-tumor effects. In hepatocellular carcinoma (HCC) models, recombinant canstatin inhibited the proliferation and migration of HepG2 and Huh7 cells and promoted apoptosis. These effects were associated with suppression of the HIF-1α/VEGF signaling pathway [37].

Tumstatin, generated from the NC1 domain of the α3 chain of Col IV, comprises several bioactive domains, each with unique anti-tumor effects [45]. Tum-5, the principal anti-angiogenic region of tumstatin, binds to the αvβ3 integrin receptor of endothelial cells. Unlike the fragments mentioned previously, Tum-5 suppresses the mechanistic target of rapamycin and eukaryotic translation initiation factor 4E-binding protein 1 (mTOR/4E-BP1) axis, removing survival signals from endothelial cells and blocking new vessel formation [46]. Delivering Tum-5 to B16F10 melanoma cells using engineered *Salmonella typhimurium* reduced tumor growth, tumor weight, CD31, and VEGF-A, while increasing survival in mice, caspase-3 activity, and apoptosis. Another subdomain of tumstatin is the T7 peptide fragment, which prevents endothelial protein synthesis and proliferation [1,47]. In a murine HCC model, combining T7 peptide with cyclooxygenase-2 and prostaglandin E2 (COX-2/PGE2) inhibitor meloxicam promotes enhanced anti-angiogenic activities in a hypoxic environment [47]. A third region, Tum185-191, acts directly on tumor cells to inhibit proliferation, stimulate apoptosis, and suppress Akt activation in A549 non-small-cell lung carcinoma [47].

Tetrastatin, derived from the NC1 domain of the α4 chain of Col IV, centers its function on tumor invasion rather than angiogenesis. Full-length tetrastatin reduced membrane type 1 matrix metalloproteinase (MT1-MMP) activation in melanoma cells, decreasing their ability to breach the ECM barrier, thereby potentially suppressing tumor growth [48]. Within this fragment, a 13-amino-acid peptide, QS-13 (QKISRCQVCVKYS), exhibits anti-angiogenic properties by binding to the αvβ3 and α5β1 integrins on endothelial cells. The peptide also impedes melanoma cell migration and proliferation by inhibiting the FAK/PI3K/Akt pathway [49].

Endotrophin is a tumor-promoting signaling fragment cleaved from the C-terminal C5 peptide of the α3 chain of Col VI (COL6A3). In contrast to the tumor-restraining functions of collagen fragments discussed above, endotrophin acts to promote rather than constrain malignancy. Endotrophin alters both cancer cells and the TME by stimulating fibrosis, inflammation, angiogenesis, metastasis, epithelial–mesenchymal transition (EMT), and resistance to anti-cancer treatment [5,50,51]. In breast cancer models, endotrophin drives tumor inflammation and angiogenesis by recruiting macrophages and endothelial cells, while additionally promoting TGF-β-dependent EMT and lung metastasis signaling, respectively [5,50,51]. More recently, Jo et al. identified a cluster of differentiation 44 (CD44) as a hepatic endotrophin receptor that activates signal transducer and activator of transcription 3 (STAT3) to promote EMT, tumor cell proliferation, and resistance to sorafenib. Notably, STAT3 upregulates *COL6A3* and CD44 expression, which reinforces endotrophin signaling and establishes crosstalk between tumor and stromal cells. This finding is significant as it addresses a gap in identifying a receptor that transmits endotrophin’s cancer-promoting signals, although CD44 should not be assumed to be the sole endotrophin receptor across all tissues [52]. Endotrophin may therefore have value as both a therapeutic target and a prognostic biomarker, but treatments remain at the preclinical stage.

Circulating fragments of Col IV (cCOL IV) in the TME have been proposed as a biomarker of metastatic disease and prognosis, as illustrated in Figure 2. A recent study by Lindgren et al. showed that circulating Col IV was elevated in metastatic breast cancer (mBC) relative to primary breast cancer (pBC) and found increased COL IV deposition in the ECM surrounding tumor cells in hepatic and osseous metastases [53]. cCOL IV was also more effective at differentiating between mBC and pBC than 15-3 (CA 15-3), a known tumor biomarker and cancer antigen. Moreover, high levels of cCOL IV predicted shorter survival, supporting their potential prognostic value as a stromal biomarker in breast cancer. However, the authors note limitations such as small cohort sizes and variation in disease stage at the time of sampling [53]. Overall, the effects of CPFs on cancer are context-dependent; some promote malignancy, whereas others exhibit anti-cancer activity depending on peptide identity, tumor type, microenvironment, and concentration.

## 4. The Effects of CPFs on Lung Health and Disease

The current literature suggests that the progression of chronic respiratory diseases is driven, in part, by disrupted collagen homeostasis, either through excess accumulation or insufficient degradation of collagens. In pulmonary health, collagen fragments derived from various fibrillar and network-forming collagen subtypes play a role by serving individually as biomarkers with prognostic value, as bioactive fragments to regulate normal processes such as angiogenesis, tissue remodeling, and inflammation, or as a combination of both. Given that the alveolar-capillary basement membrane is a collagen IV-rich structure formed by the merging of epithelial and endothelial basement membranes, CPF activity is highly relevant to normal pulmonary function [54]. This structural role, alongside the fibrillar collagen network providing interstitial support, highlights the significance of collagen fragments in pulmonary diseases, such as asthma, COPD, and cystic fibrosis.

Asthma is a chronic respiratory illness that affects at least 334 million people worldwide [55]. It is characterized by airway constriction due to inflammation, often leading to airway remodeling via deposition of type I, III, and V collagens at the bronchial submucosal level [56]. Collagen turnover yields fragments such as the C1M and MMP-cleaved type IV collagen fragment (C4M) derived from the triple-helical domain of their parent collagens Col I and Col IV, respectively. According to a study conducted by Rønnow et al., C1M was significantly upregulated in asthmatic patients compared to controls (*p* = 0.0005), particularly in patients with obesity and elevated neutrophil counts. An accompanying ovalbumin (OVA) mouse model supported this finding, demonstrating C1M levels rising with acute neutrophilic inflammation and then decreasing when neutrophil infiltration was inhibited, showing a positive relationship between neutrophil and C1M levels. C1M is not asthma-specific but instead reflects broader systemic inflammation. This distinction proposes C1M as a subtype biomarker for endotyping obese, high-neutrophil asthma rather than as a diagnostic marker for the disease [17]. Additionally, Andelic et al. measured ECM neoepitopes associated with immune cell activity, collagen formation, and degradation in 512 individuals from 256 twin pairs (312 with asthma, 200 healthy controls). C4M, a marker of collagen IV α1-chain degradation, was elevated in twins with obstructed airways, but significantly reduced in asthmatic patients. This contrast likely points to alterations in the underlying remodeling process. Elevated C4M in twins with obstructed airways indicates ongoing collagen degradation, whereas the chronic inflammatory state in asthmatic patients may reflect differences in collagen turnover tied to variations in the stage of remodeling between the two conditions [20]. Taken together, C1M and C4M illustrate the complementary and translational value of blood-based CPFs as systemic biomarkers of a local disease process.

Chronic obstructive pulmonary disease (COPD) is driven by airway inflammation and alveolar abnormalities resulting in airflow obstruction, partially characterized by neutrophil-mediated inflammation. Exacerbations contribute significantly to COPD progression, changing the proteins produced in lung ECM turnover [57,58]. One driver of this neutrophilic activity is proline–glycine–proline (PGP), a CPF released from the collagenous domains of major fibrillar and basement membrane collagens including I, III, and V through the multistep proteolytic actions of MMP-8, MMP-9, and prolyl endopeptidase [59]. The acetylated form of PGP, N-acetyl-PGP (Ac-PGP), is structurally similar to the glutamic acid–leucine–arginine (ELR)+ motif of C-X-C motif chemokine ligand 8 (CXCL8) and is proposed to activate neutrophil C-X-C motif chemokine receptor 1 and C-X-C motif chemokine receptor 2 (CXCR1/CXCR2) to drive chemotaxis [19]. Activated neutrophils release MMP-8 and MMP-9, further promoting Ac-PGP formation and a positive feedback loop of neutrophil activation, which results in chronic airway inflammation with lasting effects [60]. Furthermore, Weathington et al. determined that PGP is significantly elevated in the airways of COPD patients compared to healthy controls, giving it value as a disease-associated biomarker. Therefore, this establishes PGP and its acetylated form, Ac-PGP, as a biomarker and matrikine, respectively, in COPD [60].

Matrix metalloproteinase-degraded type IV collagen alpha-3 chain (C4Ma3) is a neoepitope generated via MMP-mediated degradation from the NC1 domain of collagen type IV alpha 3 chain (*COL4A3*) and is concentrated in the alveolar basement membrane, making it a relevant marker of basement membrane remodeling in the pulmonary system. In a prospective biomarker analysis of the Evaluation of COPD Longitudinally to Identify Predictive Surrogate Endpoints (ECLIPSE) cohort, Rønnow et al. benchmarked C4Ma3 against a COPD mortality-associated biomarker, fibrinogen, the only FDA-approved blood-based biomarker for COPD status, all-cause mortality, and across past/present exacerbations [18]. Results showed that C4Ma3 levels were elevated in COPD patients compared to never-smoking controls, and that elevated fibrinogen, relative to the published fibrinogen threshold of 350 mg/dL, did not predict all-cause mortality. Interestingly, elevated C4Ma3 was the only independent predictor of mortality, dichotomized at 5.5 ng/mL, in this subset of the population, contradicting previous ECLIPSE findings that high fibrinogen at this threshold predicts mortality for the COPD population [18]. This discrepancy may be attributed to limitations in this study, including the non-representative subset of participants chosen. Additionally, C4Ma3 needs to be further evaluated to confirm its potential as a prognostic biomarker of all-cause mortality.

Cystic fibrosis is a genetic disease caused by mutations in the cystic fibrosis transmembrane conductance regulator (CFTR) gene, which impairs chloride ion channels, resulting in upregulated sodium and fluid absorption, effectively dehydrating the airway surface liquid [61]. Along with its role in COPD, PGP may play a role in the progression of cystic fibrosis. In patients with acute pulmonary exacerbation, PGP and Ac-PGP were detected in sputum from 8 of 10 patients compared with 1 of 10 controls [61]. Additionally, PGP levels during inpatient hospitalization of individuals with acute pulmonary exacerbation declined in most patients over the course of the hospitalization, with mean PGP values dropping from 146 ng/mL to 80.0 ng/mL. This finding highlights the biomarker potential for PGP and Ac-PGP in exacerbated cystic fibrosis [61]. Cystic fibrosis is also characterized by net collagen accumulation. Using electron microscopy of age-matched tissue samples from a patient with cystic fibrosis and an age-matched control, Ulrich et al. observed a ~9-fold increase in collagen accumulation and a ~5-fold decrease in elastin within the alveolar space of cystic fibrosis patients, suggesting collagen replaces elastin during alveolar tissue remodeling [62]. Thickened septae with extensive collagen deposition were also observed in the cystic fibrosis patient [62].

## 5. Cross-Species Evidence and Emerging Functions of CPFs

This section provides an up-to-date, comprehensive overview of both biologically active and biomarker literature on collagen-derived peptides across various species and organ systems. These collagen peptide fragments may exhibit organ-,tissue-, or species-specific effects in studies using both in vivo and in vitro experimental models. However, human evidence supporting these functions remains limited for several fragmentsand represents an important area of investigation that may shed light on their therapeutic potential in disease. Table 1 summarizes fragments discussed in this review, along with additional collagen subtypes and their reported biological functions.

**Table 1:** Overview of Biological Activity of Collagen-Derived Fragments Across Different Species.

Type of Collagen	Peptide Fragment	Species	System Investigated	Biological Activity	Biological Classification	References
Type I Collagen	PGP	Mouse	Lung	Induces pulmonary vascular permeability via CXCR2	Matrikine	[63]
	C1M	Rat	Liver fibrosis models	Elevated C1M in rats with liver fibrosis	Biomarker	[64]
	CTX-1	Human	Cancer; bones	Elevated in myeloma cancer patients; linear relationship with number of bone metastases; may predict bone health in type 1 diabetic children (8–18 years old)	Biomarker	[65,66,67]
	C1158/1159	Mouse	Heart	Modulates scar maturation; stimulates angiogenesis	Matricryptin	[32]
	PICP	Human	Heart	Increased PICP correlates with increased risk of CVD; biomarker for severity of mitral valve regurgitation	Biomarker	[68,69]
	Procollagen type I N-terminal propeptide (PINP)	Human	Bone	Reflects osteoblast activity; assessment for bone formation	Biomarker	[70]
	PINP	Rat	Liver	Potential biomarker for liver fibrosis progression	Biomarker	[71]
Type II Collagen	CTX-2	Human	Cartilage	Biomarker for cartilage degradation in joint ailments	Biomarker	[72]
	Helix-II	Human	Joints	Biomarker for progression in osteoarthritis and rheumatoid arthritis	Biomarker	[73]
Type III Collagen	CTX-3	Human	Liver	Potential biomarker of fibrolysis; indicates hepatic fibrosis resolution	Biomarker	[74]
Type IV Collagen	Arresten	Bovine	Pulmonary artery endothelial cells, endothelial cells	Increased rate of apoptosis in PAE; increased apoptosis in mouse tumor	Matrikine	[75]
	Arresten	Mouse	Endothelial cells	Increased apoptosis in mouse tumor	Matrikine	[75]
	Canstatin	Human; mouse	PC-3 tumor	Inhibits human vascular endothelial cells; reduced intratumoral vascularization in mice	Matrikine	[76]
	Tumstatin	Mouse	Kidney	Reduces glomerular accumulation of collagen in diabetic conditions	Matrikine	[77]
	Tetrastatin	Mouse	Skin	Reduced tumor proliferation in melanoma	Matrikine	[48]
	Pentastatin	Human	Lung	Decreased endothelial cell barrier integrity	Matrikine	[78]
	Hexastatin	Human	Umbilical endothelial cells	Decreases endothelial cell proliferation and migration; short hexastatin peptides also implicated in adhesion regulation	Matrikine	[79,80]
Type VI Collagen	Endotrophin	Mouse; human	Adipose tissue; heart	Promotes TGF-β pathways; promotes tumor progression; associated with higher risk of heart failure	Matrikine + biomarker	[81,82]
Type XV Collagen	Restin	Bovine	Pulmonary endothelial cell line	Inhibits migration of endothelial cells; antiangiogenic properties	Matrikine	[83]
Type XVIII Collagen	Endostatin	Human	Non-small-cell lung cancer	Recombinant human endostatin + gemcitabine/cisplatin improved NSCLC response rates over chemotherapy alone; acceptable real-world safety profile	Matrikine	[84,85]

## 6. Perspectives

**Figure 3:**
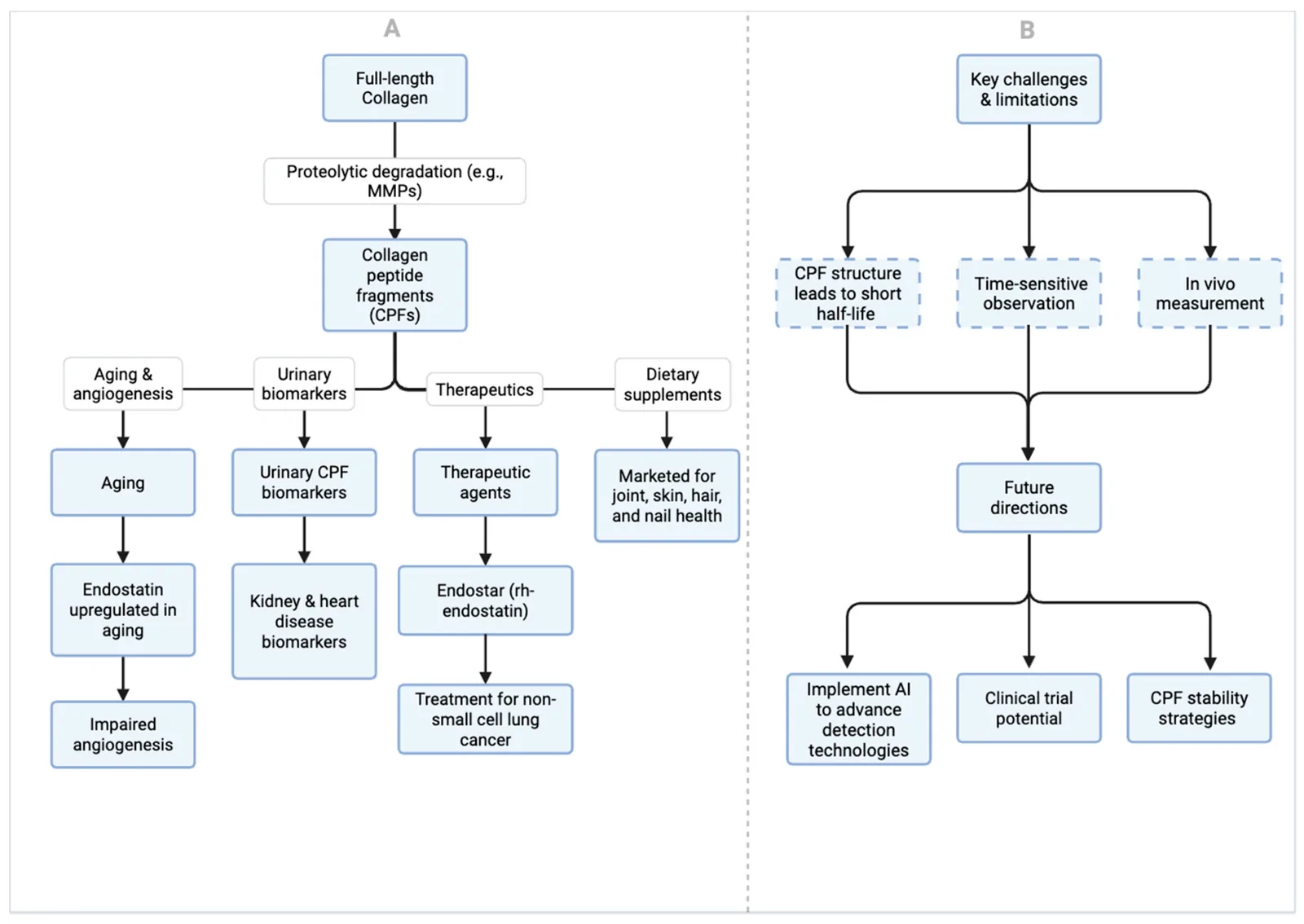
**Broader physiological, translational significance, limitations, and challenges of CPFs.** Proteolytic degradation of full-length collagen yields fragments relevant across multiple domains. (**A**) CPFs are implicated in aging, urinary diagnostics, therapeutics, and the dietary supplement industry. In aging, endostatin is upregulated [91] and may contribute to impaired angiogenesis [92,93]. Urinary CPFs serve as non-invasive biomarkers for kidney and cardiovascular disease [28,90]. Endostar is an approved pharmaceutical agent for non-small-cell lung cancer in China [86]. Additionally, collagen peptides are widely marketed in dietary supplements targeting joint, skin, hair, and nail health. (**B**) Key challenges in CPF research include their characteristically short half-life, time-sensitive observation, and difficulties measuring in vivo. Addressing these limitations will require the use of AI to advance detection technology, expansion of clinical trials among established bioactive fragments, and development of CPF stabilization strategies. Dashed borders may need further investigation and have become targets for future research directions.

The preceding sections demonstrate that CPFs cannot always be classified exclusively as passive biomarkers that report on collagen turnover or as bioactive matrikines that actively modulate the tissue microenvironment. Some CPFs serve both roles, such as endotrophin, which functions as a bioactive matrikine and as a disease-associated biomarker. While these fragments are convenient to analyze in separate contexts regarding health and disease, the biological reality is that a single fragment can simultaneously hold various beneficial and/or adverse roles in multiple different systems. Recognizing these multifaceted functions provides a comprehensive understanding of potential therapeutic targets outlined in Figure 3.

Endostatin is among the most clinically advanced collagen-derived anti-angiogenic fragments. Different forms of recombinant human endostatin have undergone clinical evaluation, including in combination with chemotherapy for non-small-cell lung cancer [84]. Endostar is a modified recombinant human endostatin containing an additional nine-amino-acid sequence (MGGSHHHHH) at its N-terminus, and this modification has been associated with improved protein stability compared with earlier recombinant endostatin forms and may contribute to its enhanced therapeutic activity [86,87]. Endostar was approved in China as an anti-angiogenesis add-on therapy for advanced non-small-cell lung cancer to make platinum-based chemotherapy treatments more effective. However, the pharmacological agent has not been approved for use by the FDA in the United States [87].

The investigation of Endostar applications in other solid cancers, such as osteosarcoma, cervical, liver, and gastrointestinal cancers, is in its early stages. Notably, in cervical cancer, the chemoradiotherapy response rate increased from 54.55% to 74.47% with the addition of Endostar, while also decreasing related tumor markers and increasing survival within three years [88]. In addition to blocking angiogenesis, Endostar may potentially be utilized as an immune-sensitizing treatment to improve tumor recognition in CD8+ cytotoxic cells. However, this effect has only been studied in vitro using cultured A549 and NCI-H1299 lung cancer cells, and additional studies in human and animal tumor models are needed [87]. Endostar offers an alternative approach in targeting the interconnected aspects of cancer, specifically the vascular supply. Additional trials outside of Chinese populations are needed before Endostar can be considered a broadly applicable co-treatment across cancers.

Another advancement in CPF-based therapies lies in utilizing urinary collagen fragments, as mentioned in the cardiovascular section. Devos et al. investigated the influence of nine abundant urinary α1(I)-derived peptide sequences on endothelial cell migration. Although none of the urinary peptides altered migration at their physiological concentrations (100 nM), the NGDDGEAGKPGRPGERGPOGO fragment reduced Col I-induced migration of human umbilical vascular endothelial cells (HUVEC) by nearly 50%; the mean number of migrated endothelial cells was 30.58 cells with full-length Col I treatment alone, compared to 15.92 cells with the addition of the fragment. This highlights the fragment’s potential regulatory role in Col I-induced endothelial cell migration [89].

However, the proposed mechanism requires experimental confirmation, as it is solely supported by AlphaFold 3 modeling and phosphoproteomics. Currently, this fragment and many urinary collagen fragments are used as biomarkers in kidney and cardiovascular disease [90]. Interestingly, there is potential for urinary collagen fragments to have clinical applications, as mentioned in Figure 3.

An important aspect of determining the clinical implications of CPFs is assessing their efficacy in comparison with established pharmaceutical agents. However, comparative evidence remains limited. In a recent study using a zebrafish xenograft model, Jin et al. compared Endostar with bevacizumab and reported that both treatments inhibited the growth of A549 lung cancer xenografts [94]. This study highlights the need for researchers to compare CPFs with known pharmaceutical agents; as such, comparative data are necessary for clinical translation.

Despite their emerging biological and clinical relevance, as mentioned in Figure 2, several important challenges limit the study and interpretation of CPFs. Limitations include ambiguity in the tissue of origin of circulating fragments, the nonspecificity of collagen turnover as a disease signal, the short circulating half-lives of CPFs, and difficulties in reliable in vivo measurement.

Collagen is ubiquitously expressed and distributed throughout the human body, and all collagen degradation across tissues releases CPFs into systemic circulation. Consequently, a fragment measured in serum integrates collagen turnover across the whole body and cannot always be attributed to a single organ. This limitation plays a significant role in patients who present with comorbidities. López et al. note that blood biomarkers of collagen metabolism are not cardiac specific, which raises challenges in relating CPF measurements to the structural alterations of the myocardial collagen network [95].

Another limitation arises from the physical properties of the fragments themselves. Once collagen undergoes proteolysis, the resulting fragments are vulnerable to further proteolytic degradation into smaller peptides, leaving only a narrow temporal window to study these fragments. Endostatin, for example, has a short half-life of approximately 1–2 h, and fusion to an IgG Fc domain increased its half-life to longer than a week [96].

Finally, CPF measurements are constrained by the assays used to obtain them. Rubiś et al. investigated 22 assays across 89 studies and determined that CPF measurements were highly variable, especially between assays from different manufacturers. In their report, values spanned PIIINP 0.06–11,800 μg/L, PICP 0.006–1265 μg/L, CITP 0.3–5450 μg/L, and PINP 0.15–80 μg/L [97].

Collagen fragments were long thought to be biologically inactive degradation products. Studies within this field have uncovered the biological activity of many peptide fragments, and although they are typically unstable due to their short half-lives, there is therapeutic potential for stabilized CPFs.

## 7. Conclusions

In summary, CPFs are involved in many biological processes and demonstrate high therapeutic and biomarker potential. However, many gaps in our understanding of the mechanisms of action of known CPFs remain significant limitations. Additionally, the short half-life of peptide fragments is an obstacle in investigating their true biological potential in health and disease. Collagens are very stable proteins due to their triple-helical chain structure. Peptide fragments, derived from a single chain, lack the structural properties required to remain stable, making them more susceptible to further degradation. In the context of biological function, reduced stability tends to lead to more rapid degradation and shorter half-lives, making it challenging to study peptide fragments in vivo. Finding creative ways to measure peptide fragments, such as synthesizing a stable mimetic fragment or stabilizing an existing fragment by adding amino acids, is essential for understanding the biological mechanisms and activities and advancing the mechanistic and translational understanding of CPFs.

Our previous review [4] underscored the importance of CPFs in cardiovascular health and disease. This review builds upon the previous article by expanding the discussion of peptide fragments in cardiovascular, cancer, and pulmonary systems in health and disease. On that basis, this review highlights the broad and underexplored domain of CPFs. Extending investigations into structure–function relationships and enhanced stability strategies will be necessary to realize the translational potential of CPFs. As technologies for peptide fragment detection advance, CPFs may emerge as vital biomarkers and targeted therapeutics across many pathophysiological conditions.

## Data Availability

No new data were created or analyzed in this study. Data sharing is not applicable to this article.

## Abbreviations

The following abbreviations are used in this manuscript:

Akt	Protein kinase B
Ac-PGP	N-acetyl-PGP
C1M	MMP-mediated type I collagen degradation
C4M	MMP-cleaved type IV collagen fragment
C4Ma3	Matrix metalloproteinase-degraded Type IV collagen alpha-3 chain
CITP	Carboxyl-terminal telopeptide of type I collagen
COL1A1	Collagen type I alpha 1 chain
COL3A1	Collagen type III alpha 1 chain
COL4A3	Collagen type IV alpha 3 chain
CPF	Collagen peptide fragment
ECLIPSE	Evaluation of COPD Longitudinally to Identify Predictive Surrogate Endpoints
EMT	Epithelial–mesenchymal transition
FAK	Focal adhesion kinase
HF	Heart failure
HIF	Hypoxia-inducible factor
MI	Myocardial infarction
MMP	Matrix metalloproteinase
PICP	Carboxy-terminal propeptide of procollagen type I
PINP	Procollagen type I N-terminal propeptide
